# Maternal Psychosocial Stress during Pregnancy and Placenta Weight: Evidence from a National Cohort Study

**DOI:** 10.1371/journal.pone.0014478

**Published:** 2010-12-31

**Authors:** Marion Tegethoff, Naomi Greene, Jørn Olsen, Andrea H. Meyer, Gunther Meinlschmidt

**Affiliations:** 1 Division of Clinical Psychology and Psychotherapy, Department of Psychology, University of Basel, Basel, Switzerland; 2 Department of Neurobehavioral Genetics, Institute of Psychobiology, University of Trier, Trier, Germany; 3 Department of Epidemiology, School of Public Health, University of California Los Angeles, Los Angeles, California, United States of America; 4 Department of Epidemiology, The Danish Epidemiology Science Centre, Institute of Public Health, University of Aarhus, Aarhus, Denmark; 5 Department of Applied Statistics in Life Sciences, Department of Psychology, University of Basel, Basel, Switzerland; 6 National Centre of Competence in Research “Swiss Etiological Study of Adjustment and Mental Health (sesam),” Basel, Switzerland; The University of Adelaide, Australia

## Abstract

**Background:**

To study in a large-scale cohort with prospective data the associations between psychosocial stress during pregnancy and placenta weight at birth. Animal data suggest that the placenta is involved in stress-related fetal programming.

**Methodology/Principal Findings:**

We defined *a priori* two types of psychosocial stress during pregnancy, life stress (perceived burdens in major areas of life) and emotional symptoms (e.g. anxiety). We estimated the associations of maternal stress during pregnancy with placenta weight at birth, controlled for length of gestation, by predicting gestational age- and sex-specific z-scores of placenta weight through multiple regression analysis, adjusted for potential confounders (N = 78017 singleton pregnancies). Life stress (per increase in stress score by 1, range: 0–18) during pregnancy was associated with increased placenta weight at birth (z-score, reported in 10^−3^; B, 14.33; CI, 10.12–18.54). In contrast, emotional symptoms during pregnancy were not associated with placenta weight at birth.

**Conclusions/Significance:**

Maternal life stress but not emotional symptoms during pregnancy was associated with increased placenta weight at birth; yet, the association-estimate was rather small. Our results may contribute to a better understanding of the role of the placenta in the regulation of intrauterine processes in response to maternal stress.

## Introduction

Changes in placental growth have been associated with adverse health outcomes for mother and fetus [1], [2]. Moreover, an adverse intrauterine environment has repeatedly been associated with an increased risk of cardiovascular and metabolic diseases and premature mortality in adult life [3]. This phenomenon, referred to as fetal programming, could have profound impact on public health strategies for the prevention of major diseases [4].

Any disturbances in the maternal compartment, for example due to environmental challenges, which impact on the fetus, will be transmitted across the placenta. There is an increasing awareness that the placenta responds to perturbations in the maternal compartment with a wide range of structural and functional alterations, including changes in placental growth [4], [5], [6].

The idea that psychosocial stress during pregnancy (‘maternal stress’) influences the fetus, with long-term consequences into adult life, has been repeatedly corroborated [7]. However, the impact of maternal stress on placental growth, a putative key mediator in fetal programming by maternal stress, has not yet been studied.

Our primary objective was to estimate in a population-based cohort with prospective data, the association between maternal stress during pregnancy and placenta weight. We *a priori* defined two common forms of maternal stress, self-reported life stress in terms of burdens in major areas of life, and emotional symptoms [8]. The separation of these two major subtypes of maternal stress is based on their possible differential effects on the fetus [9] and the stressor-specificity of biological responses [10], [11], which may differentially affect the placenta via different biological pathways [12].

## Methods

### Ethics statement

All participants gave written informed consent and the Danish National Committee for Biomedical Research Ethics, Copenhagen, approved the study.

### Study cohort

The present study is based on data of the Danish National Birth Cohort [13]. Between 1996 and 2002, the Danish National Birth Cohort enrolled 101,042 pregnancies into a nationwide longitudinal study that aims at following up the offspring cohort for decades. We considered as eligible all pregnancies with live singleton births.

### Assessment of psychosocial stress

We studied two *a priori* defined types of maternal stress, (i) negative emotional states (‘emotional symptoms’), and (ii) psychological distress caused by burdens in major areas of life (‘life stress’). We obtained information on maternal stress during pregnancy from a computer-assisted interview taken around 30 weeks gestation. We defined life stress and emotional symptoms as previously described [8], [14]: The applied inventory on emotional symptoms during pregnancy is a modified version of the short version of the Symptom Checklist (SCL)-8d [15] and covers self-reported maternal feelings (e.g. anxiety, nervousness, for detailed description of items, see Table 1). Items have been selected from The Symptom Check List-90R [16] and The General Health Questionnaire [17], to cover the most frequent emotional symptoms in adult women. Response categories were adjusted to fit the telephone interview conditions (see below). To prevent somatic confounding due to physical conditions during pregnancy, we only included items related to emotional symptoms, but none related to somatic symptoms. Emotional symptoms were assessed by nine questions, each covering the time period since the beginning of pregnancy. Answers (no = 0, a little  = 1, a lot  = 2) were added up into a score (range: 0 to 18). We validated the scale according to several types of validity [18]. Internal consistency among items was satisfactory (Cronbach's alpha  = 0.75). Construct validity was determined using two external validations, demonstrating that the emotional symptoms scale clearly distinguished between pregnant women (i) suffering versus not suffering from a mental disorder during pregnancy (two-sample t test with equal variances: *t*_1,63385_ = −45.64; *P*<0.001; mean emotional symptoms score (95% confidence interval, CI) in sufferers and non-sufferers: 6.97 (6.68–7.26) and 2.72 (2.70–2.74), respectively), and (ii) having versus not having consulted a psychologist/psychiatrist in the past (*t*_1,63799_ = −53.08; *P*<0.001; mean emotional symptoms score (95% CI) in consulters and non-consulters: 4.73 (4.63–4.83) and 2.64 (2.62–2.66), respectively). Moreover, we tested the scale's relationship with the Symptom Check List-90R in a separate sample of pregnant women (*N* = 64), relating the selected items with the Symptom Check List-90R global severity index (Spearman-Rho correlation: *r* = 0.917; *P*<0.001). The applied inventory on life stress during pregnancy focuses on whether the women felt burdened in major areas of life, including financial circumstances, housing, work, relations, pregnancy, and health (Table 1), based on the major categories of the Life Events Questionnaire [19]. We tested the inventory's relationship with a standard measure of daily hassles [20] in a separate sample of pregnant women (*N* = 65), relating nine comparable items with the total score (Spearman-Rho correlation: *r* = 0.899; *P*<0.001). Life stress was assessed by nine questions, each covering the time period since the beginning of pregnancy. Answers (no  = 0, a little  = 1, a lot  = 2) were added up into a score (range: 0 to 18). We dealt with up to two missing answers per score by using person-specific mean substitution. Women with more missing answers were excluded. In the analysis, we considered life stress and emotional symptoms as continuous independent variables.

**Table 1:** Items used to assess life stress and emotional symptoms.

Life stress		Emotional symptoms	
Have you felt burdened during pregnancy by any of the things I am going to ask now? You may answer: no, a little or a lot.		Now I am going to ask you how you have been feeling during pregnancy. You may answer: no, a little or a lot.	
Have you been burdened by…		Have you felt…	
1.	…financial circumstances?	1.	…scared for no reason?
2.	…your housing situation?	2.	…hopeless about the future?
3.	…your work situation?	3.	…constantly under strain?
4.	…the relationship to your partner?	4.	…nervous or shaky inside?
5.	…the relationships to your family and friends?	5.	…blue?
6.	…your pregnancy?	6.	…easily annoyed or irritated?
7.	…own diseases?	7.	…everything was an effort?
8.	…disease of your partner, family members or close friends?	8.	…tense or keyed-up?
9.	…other things?	9.	…that everything was getting on top of you?

### Outcome measures

Placenta weight at birth is a summary measure of placenta growth and development throughout pregnancy [21]. Placenta weight was determined by trained midwives according to standard procedures issued by the National Board of Health. Each placenta was weighed with a regularly calibrated digital scale between 15 to 20 minutes after delivery, including membranes and umbilical cord. The placenta was neither washed nor dried. Information on placenta weight was extracted, together with information on other obstetric outcomes, from the Danish National Hospital Register including the Medical Birth Registry, which is linked to the Danish National Birth Cohort database and covers all deliveries in Denmark. The hospital registers provide accurate reporting of obstetric outcomes. To account for length of gestation in the placenta weight at birth, we calculated the sex-and gestational age (in days)-specific z-scores of placenta weight (standardized residuals from the regression of placenta weight at birth on gestational age at birth [linear and quadratic terms] separately for males and females of the study sample) [22]. We included placenta weight as continuous dependent variable.

### Statistical analyses

We performed the descriptive analyses of maternal and infant demographic, anthropometric, and clinical baseline characteristics and of independent and dependent variables by calculating frequencies and percentages of the discrete variables. We calculated means and standard deviations for symmetrically distributed variables and medians and ranges for variables with non-symmetrical distributions, as distinguished by visual inspection of data plots.

To determine the associations of life stress and emotional symptoms during pregnancy with placenta weight at birth, we conducted linear regression analyses. To obtain less biased estimates, we adjusted our model *a priori* for several well established predictors of placenta weight that may confound or suppress effects, including maternal age, infant sex, parity, maternal pre-pregnancy body mass index, occurrence of hypertension and diabetes during pregnancy, and smoking status, with the categories indicated in Table S1 [21], [23], [24], [25]. We obtained information on most of these variables from 3 computer-assisted telephone interviews at approximately 12 and 30 weeks gestation and 6 months postpartum; information on infant sex was retrieved from the Medical Birth Registry. We inspected residual plots to verify linearity, normality, and homoscedasticity assumptions and ensured that multicollinearity between covariates was generally low based on variance inflation factors. We excluded 208 newborns with biologically unrealistic or extreme values in obstetric outcomes such as the lowest and uppermost 0.1% of placenta weight, and for body weight, body length, abdominal circumference, and head circumference, all values lying outside the range of their gestational age-specific mean +/− 3 standard deviations, according to previously established growth charts [26], [27].

Altogether 4842 singletons were born to mothers who contributed more than one pregnancy to the study. To correct for possible dependence between birth outcomes in these infants, all standard errors were calculated with use of the clustered sandwich estimator. Moreover, we repeated all analyses including only the first pregnancy of each woman in the cohort to control for previous reproductive experiences [28].

We report unstandardized (B) regression coefficients, including 95% CIs, standardized (beta) regression coefficients, and, for illustrative purposes, *p*-values for each of the predictors (life stress and emotional symptoms). To additionally test whether the regression coefficients of the predictors were robust, we cross-validated each adjusted regression model by using the Chow-test [29]: We split the total sample into two random parts, amounting to 75 and 25% of the total sample, and created a new variable that indicated to which sub-sample each case belonged. We then computed two interaction terms by multiplying this grouping variable by each of the two predictors. We re-performed the separate multiple regression analyses in the total sample, including the grouping variable as well as the interaction terms. A non-significant *P*-value of the interaction term indicates that the assumption of no interaction, meaning no difference between the 75 and 25% sub-samples, is compatible with data.

To further explore whether the identified associations were present across all levels of socioeconomic status, we performed *a posteriori* analyses, in which we repeated the calculations, stratifying according to the pregnant women's socioeconomic status (based on the mother's occupation [30]).

Moreover, to determine whether the relationship between life stress and gestational age-adjusted placenta weight could be explained by related changes in gestational age-adjusted birth weight, we performed mediation analysis using a bootstrapping procedure (200 repetitions) developed by Preacher and Hayes [31], using standard estimators. Sex-and gestational age (in days)-specific z-scores of birth weight were calculated according to the procedure outlined above for placenta weight (standardized residuals from the regression of fetal weight at birth on gestational age at birth [linear and quadratic terms] separately for males and females of the study sample).

All tests were two-tailed and we set the level of significance at .05. We dealt with loss to follow-up and missing data (i) in the exposure scores and outcome variables by restricting analyses to mother-newborn pairs with complete data, and (ii) in the covariates by including an extra category for those with missing information in the analyses (see Table S1).

For statistical analyses, we used Stata software (version 10.0 SE; Stata Corporation, College Station, Texas).

## Results

### Study cohort descriptives

**Figure 1:**
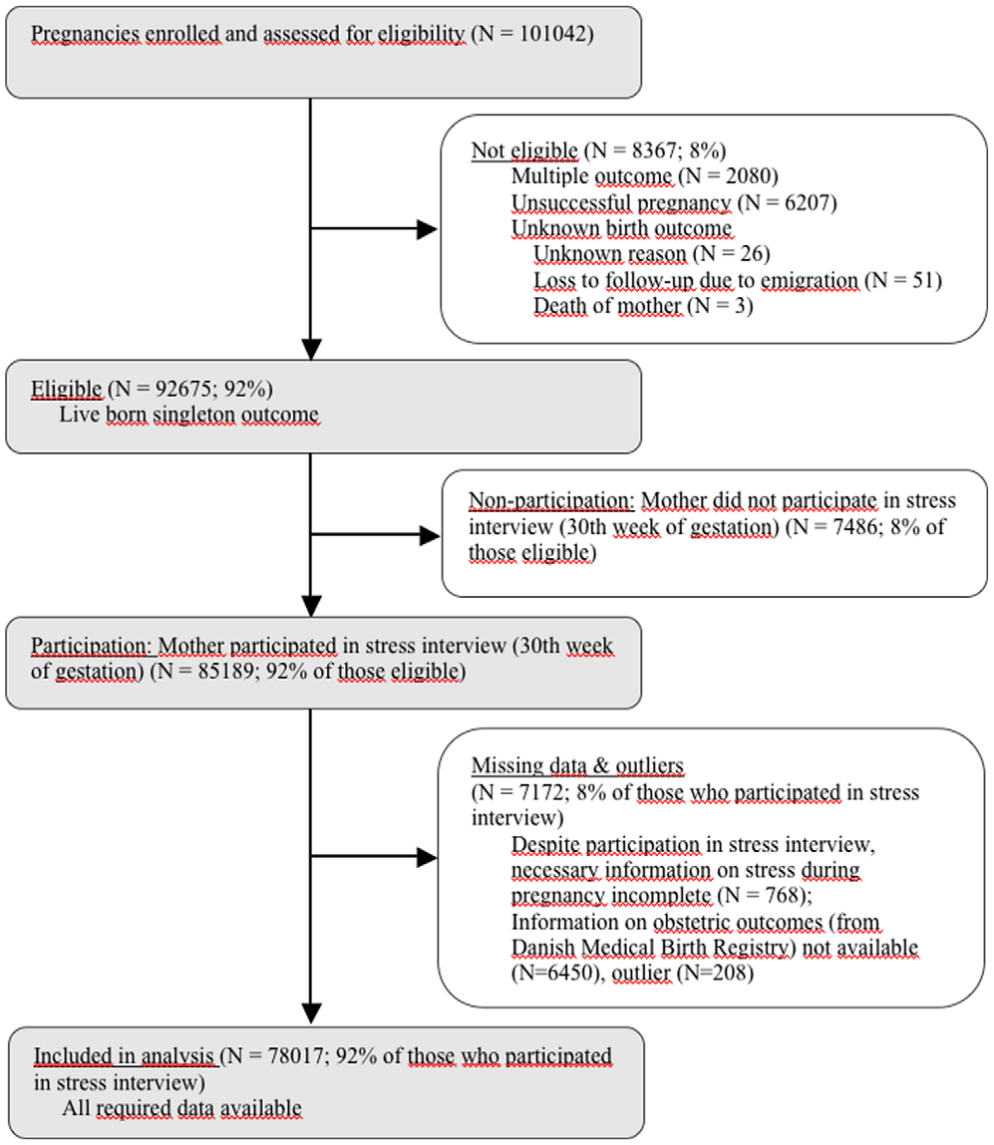
Flowchart of Study Participants.

Out of the 101042 pregnancies initially enrolled in the Danish National Birth Cohort (approximately 30% of all Danish births in the study period and 60% of those invited to the study [13]), we considered 92676 (92%) eligible for participation. Out of these, 85189 mothers (92%) completed the required interview at 30 weeks of gestation. Complete data on maternal stress during pregnancy, placenta weight, length of gestation, and fetal sex were available for 78017 (92%) of the remaining mother-newborn pairs (Figure 1). Table S1 gives details on maternal and infant baseline characteristics, including the stress scores, placenta weight and covariates under study.

### Multiple Regression Analyses

Table 2 presents the results of the multiple regression analysis reflecting the associations of life stress and emotional symptoms with the gestational age- and sex-specific z-score of placenta weight at birth. After adjustment for maternal age, infant sex, parity, pre-pregnancy body mass index, hypertension, gestational diabetes, and smoking, higher life stress during pregnancy was significantly associated with a higher z-score of placenta weight at birth. There was no significant association between emotional symptoms during pregnancy and the z-score of placenta weight at birth. The adjusted model was highly significant (*p*<0.001) but explained only 3.5% of the variance in the outcome measures.

**Table 2:** Adjusted and Unadjusted Regression Coefficients for Placenta Weight at Birth, Corrected for Length of Gestation^A^ (Outcome), According to Life Stress and Emotional Symptoms During Pregnancy (Predictors) (*N* = 78017).

	Parameter estimates of the crude^B^ model			Parameter estimates of the adjusted^C^ model		
	*B [95% CI for B]*	*Beta* D	*p*	*B [95% CI for B]*	*Beta* D	*p*
**Placenta weight (ZS)**						
Life stress	22.21 [17.95, 26.47]	0.043	<0.001	14.77 [10.56, 18.98]	0.029	<0.001
Emotional symptoms	−2.12 [−5.32, 1.08]	−0.006	0.193	−1.20 [−4.37, 1.97]	−0.003	0.458
*Crude Model: F(2,73174) = 63.13, P<0.001, R^2^ = 0.002*						
*Adjusted Model: F(18, 73174) = 151.79, P<0.001, R^2^ = 0.035*						

*Note.* Life stress and emotional symptoms are continuous variables. CI  =  confidence interval.

A As indicated by the gestational age- and sex-specific z-scores of placenta weight at birth. For convenience, the unstandardized regression coefficient estimates (B) (and their 95% CI) for z-standardized dependent variables are presented in [*10^−3^].

B Crude model provided in support of transparency.

C Model adjusted for maternal age, infant sex, pre-pregnancy body mass index, parity, hypertension, gestational diabetes, and smoking.

D To provide statistical values, which allow comparison of results between separate regression analyses, standardized regression coefficient estimates (beta) were calculated in addition to the unstandardized regression coefficient estimates (B). As the clustered variance estimation procedure does not provide betas, for illustrative purposes, betas were calculated with the robust variance estimation procedure.

Cross-validation confirmed the stability of the regression coefficients of life stress and emotional symptoms across the two random sub-samples (interaction sub-sample group x predictor: all *p*>0.05). When we repeated adjusted analyses using only the women's first pregnancies, regression coefficients were of similar magnitude as those presented in Table 2. Estimates of the associations of life stress and emotional symptoms during pregnancy, with placenta weight at birth not corrected for length of gestation, are provided online (see Table S2). In addition, results of the secondary analyses stratified according to the women's socioeconomic status are provided as supplemental material (see Table S3).

Mediation analysis revealed that the direct association between life stress and placenta weight accounted for 56.7% of the observed total relationship between life stress and placenta weight (B = 8.53, CI  = 5.29–11.77, z-score, reported in 10^−3^), while 43.3% of the observed total relationship between life stress and placenta weight was mediated by changes in birth weight (B = 6.24, CI  = 3.96–9.09, z-score, reported in 10^−3^).

## Discussion

Our main finding was that life stress but not emotional symptoms was moderately associated with placenta weight at birth controlled for length of gestation, indicating that the placenta may play a role in the regulation of intrauterine processes in response to certain maternal adversities. Our findings corroborate evidence from animal studies showing that stress may affect the placenta [32]. In line with our results, previous human studies suggest that the placenta has the potential for compensatory growth in response to other adverse pregnancy exposures [33], [34], [35].

We do not know what biological processes mediate the association between life stress and placenta weight, but the insulin-like growth factor system and the placental growth hormone system have an impact on placental growth [36], [37], [38], [39], and an increased production of insulin-like growth factor hormones and placental growth hormone has been observed in an unfavorable intrauterine environment [40], [41]. Furthermore, the stimulation of the insulin-like growth factor system may be subject to a placental protein that is highly expressed early in pregnancy, the pregnancy-associated plasma protein-A (PAPPA) [42], which can be stimulated by stress-activated signaling pathways [43]. Interestingly, recent data also suggest a role of cytokines as potential mediators between stress during pregnancy and placental growth. For example, elevated psychosocial stress appears to be linked to lower interleukin 10 (IL10) concentrations in early pregnancy [44], while il10 deficiency in mice increased placental size by 28 percent [45]. Finally, in male mice, psychosocial stress early in pregnancy significantly increased expression of peroxisome proliferator-activated receptor alpha (PPARα), insulin-like growth factor binding protein 1 (IGFBP-1), hypoxia-inducible factor 3a (HIF3), and glucose transporter 4 (GLUT4) [32], suggesting a potential epigenetic mechanism whereby maternal stress may directly affect placenta gene expression patterns.

The fact that the relationship between life stress and placenta weight accounts for only 3–4% of the variance suggests a potential role for moderators affecting the strength of association between maternal stress during pregnancy and placenta weight, for example genomic variation. Indeed, several genetic factors have been identified that are directly associated with placental growth, such as the gene coding for the growth factor receptor-bound protein 10 (*GRB10*) [46] and pleckstrin homology-like domain, family A, member 2 (*PHLDA2*) [47]. While inactivation of the maternally-inherited copy of the *GRB10* and of *PHLDA2* results in placental growth enhancement [46], [47], the potential of these genes to negatively regulate placenta growth suggests them at the same time as candidate resilience factors to protect the placenta against stress-induced overgrowth. Besides this, recent evidence indicates that polymorphisms in the gene coding for acid phosphatase 1 (*ACP1*) are linked with the association between environmental factors and feto-placental co-development [48]. This provides the hypothesis that the genetic make-up may also moderate the association between maternal stress and placenta growth. But also non-genomic factors may act as potential moderators. For example, the above-mentioned work by Coussons-Read and colleagues [44] points to the importance of social support which may act as resilience factor against stress-related changes in cytokine production that may increase occurrence of poor pregnancy outcomes [49]. In sum, several biological mechanisms are candidate mediators or moderators of the association between maternal stress and placenta weight. These should be scrutinized in future studies.

Our findings may have different implications. First, the placenta plays a major role in fetal growth [50], and fetal growth depends on placenta weight across the entire range of the growth spectrum [51], [52]. Hence, increased placenta weight after maternal life stress may compensate for reduced fetal growth or lead to increased fetal growth. In line with the latter, we have recently shown in the same sample that maternal life stress was associated with increased offspring weight at birth [14]. However notably, 56.7% of the association between life stress and placenta weight was independent of related variation in birth weight. This suggests that the relationship between life stress and placenta weight is predominantly reflective of a process independent of fetal growth. Second, while maternal psychosocial stress may also be one important risk factor for preterm birth [53], and corticotropin releasing hormone (CRH), which is highly expressed in the placenta [54] in concentrations positively related to placental mass [55], is involved in the timing of parturition [56], the role of placental CRH as a putative physiological mediator bridging maternal stress and reduced length of gestation remains weak [57], [58]. Third, high placenta weight has previously been shown to be a risk factor for hypertension in the offspring and maternal cancer [1], [2], indicating that in concert with other risk factors, increases in placenta weight related to common stress may be clinically relevant.

Important strengths of this study include the prospective data collection for a total of 78017 mother-newborn pairs allowing to detect even subtle associations, the linkage of a comprehensive medical birth registry providing data which we expect to be without systematic measurement errors, and the definition of maternal stress with a focus on everyday occurrence (rather than rare disasters), which has major relevance within the general population. Moreover, we were able to adjust our analyses for a number of major potential confounders. Therefore, we believe it is unlikely that residual confounding has biased our results but our findings need to be corroborated in an independent data set. Further, we verified the stability of our results by cross-validation and repeated all analyses including only the first pregnancy of each woman in the cohort to control for previous reproductive experiences [28], which resulted in no relevant change in the estimates. Finally, life stress and emotional symptoms scores in the study cohort were comparable to previously reported degrees of life stress and emotional symptoms in adults [15], [19]. However, the here reported scores were lower than depressive symptoms scores assessed by the Edinburgh Postnatal Depression Scale, in pregnant women [59]. This may be due to a lower sensitivity of the response categories of our scales.

There are also limitations. First, we did not have data on the timing of stress exposure, which has been shown to play a role in the relation between maternal stress and placental changes in animals [32]. However, both life stress and emotional symptoms most likely reflect rather chronic stress states, which are often impossible to time precisely. Second, we used information on placenta weight at birth, controlling for length of gestation by calculating gestational-age adjusted z-scores [22], but we did not have data on growth rates for any time periods of gestation. To obtain more detailed data on the relationship between perceived maternal stress and placental growth trajectory, future studies should use repeated placental ultrasound measures [60]; this is, however, almost impossible within a population-based cohort.

Of all eligible mother-newborn pairs, 92% participated in the required interview, and of these, we included 92% in our analyses. However, on the basis of the good retention rate and the high percentage of complete data, we think it is unlikely that loss of mother-newborn pairs has introduced relevant bias.

As the interview in which information on maternal stress was assessed was taken between 6 and 7 months of gestation, no information on maternal stress was available for those pregnancies that terminated before the interview and, hence, these mother-newborn pairs were excluded from our analyses. Consequently, we have limited data on extreme preterm births. Moreover, the relationship between life stress and gestational age-adjusted placenta weight might not be generalized to pregnant women with a low socioeconomic status. The reasons should be scrutinized in future studies.

Future studies are also needed to learn about the underlying physiological mechanisms and clinical relevance of the observed associations. As placenta weight only gives crude insights into the role of the placenta in fetal programming of long-term health [61], stress-related alterations in placental structure and function should be addressed in more detail.

In this cohort, common life stress but not emotional symptoms during pregnancy was associated with moderately increased placenta weight at birth controlled for length of gestation.

## Supporting Information

Table S1 Characteristics of the Study Cohort of Mother-Newborn Pairs. Note: d =  day. A If variables were symmetrically distributed. B If variables were not symmetrically distributed.
(0.06 MB DOC)

Table S2 Adjusted and Unadjusted Regression Coefficients for Absolute Placenta Weight at Birth (Outcome), According to Life Stress and Emotional Symptoms During Pregnancy (Predictors) (N = 78017). Note. Life stress and emotional symptoms are continuous variables. CI =  confidence interval. A Crude model provided in support of transparency. B Model adjusted for maternal age, infant sex, pre-pregnancy body mass index, parity, hypertension, gestational diabetes, and smoking. C To provide statistical values, which allow comparison of results between separate regression analyses, standardized regression coefficient estimates (beta) were calculated in addition to the unstandardized regression coefficient estimates (B). As the clustered variance estimation procedure does not provide betas, for illustrative purposes, betas were calculated with the robust variance estimation procedure.
(0.03 MB DOC)

Table S3 Adjusted and Unadjusted Regression Coefficients for Placenta Weight at Birth, Corrected for Length of Gestation (Outcome)A, According to Life Stress and Emotional Symptoms During Pregnancy (Predictors), Stratified According to Socioeconomic Status (N = 78017). Note. Life stress and emotional symptoms are continuous variables. CI =  confidence interval. SES  =  socioeconomic status. A As indicated by the gestational age- and sex-specific z-scores of placenta weight at birth. For convenience, the unstandardized regression coefficient estimates (B) (and their 95% CI) for z-standardized dependent variables are presented in [*10^−3^]. B Crude model provided in support of transparency. C Model adjusted for maternal age, infant sex, pre-pregnancy body mass index, parity, hypertension, gestational diabetes, and smoking. D To provide statistical values, which allow comparison of results between separate regression analyses, standardized regression coefficient estimates (beta) were calculated in addition to the unstandardized regression coefficient estimates (B). As the clustered variance estimation procedure does not provide betas, for illustrative purposes, betas were calculated with the robust variance estimation procedure.
(0.04 MB DOC)
