# Supplementary material for: Maternal Psychosocial Stress during Pregnancy and Placenta Weight: Evidence from a National Cohort Study
Source: PLoS One. 2010 Dec 31;5(12):e14478. doi: 10.1371/journal.pone.0014478 (PMC3013108; doi:10.1371/journal.pone.0014478)
Supplement: Table S1 — Characteristics of the Study Cohort of Mother-Newborn Pairs. Note: d = day. A If variables were symmetrically distributed. B If variables were not symmetrically distributed. (0.06 MB DOC) [file pone.0014478.s001.doc]

**Supplemental Digital Content 1 (Table S1):**Characteristics of the Study Cohort of Mother-Newborn Pairs.

| Total study population (*N*) | 78017 |
| --- | --- |
| ***Continuous variables: Mean (standard deviation)***^A^ ***/ Median (range)***^B^ | |
| *Maternal baseline characteristics* | |
| Maternal height (cm) | 169 (143-198) |
| Maternal prepregnancy weight (kg) | 67.2 (12.8) |
| Maternal life stress during pregnancy (score) | 1 (0-16) |
| Maternal emotional symptoms during pregnancy (score) | 2 (0-18) |
| *Obstetric and anthropometric birth measures* | |
| Length of gestation (d) | 280.59 (10.42) |
| Birth weight (g) | 3614.11 (519.30) |
| Placenta weight (g) | 666.06 (144.63) |
| ***Discrete variables: N (%)*** | |
| *Demographics* | |
| *Maternal age (years)* |  |
| <27 | 16534 (21.19) |
| 27-29 | 20989 (26.90) |
| 30-32 | 19519 (25.02) |
| >32 | 20975 (26.88) |
| *Socioeconomic status* | |
| High | 38124 (48.87) |
| Medium | 27003 (34.61) |
| Low | 6327 (8.11) |
| Unknown | 6563 (8.41) |
| *Infant sex* | |
| Male | 39818 (51.04) |
| Female | 38199 (48.96) |
| *Biomedical information* | |
| *Parity* |  |
| Primiparous | 34463 (44.17) |
| Multiparous | 40137 (51.45) |
| Unknown | 3417 (4.38) |
| *Pre-pregnancy body mass index (kg/m^2^)* |  |
| ≤20 | 12190 (15.63) |
| >20-22.5 | 23548 (30.18) |
| >22.5-25 | 17399 (22.30) |
| >25 | 20312 (26.04) |
| Unknown | 4568 (5.86) |
| *Smoking* |  |
| Yes | 20374 (26.12) |
| No | 43859 (56.22) |
| Unknown | 13784 (17.67) |
| *Gestational hypertension* |  |
| Yes | 8900 (11.41) |
| No | 53095 (68.05) |
| Unknown | 16022 (20.54) |
| *Gestational diabetes* |  |
| Yes | 1617 (2.07) |
| No | 59574 (76.36) |
| Unknown | 16826 (21.57) |
