# Supplementary material for: Maternal Psychosocial Stress during Pregnancy and Placenta Weight: Evidence from a National Cohort Study
Source: PLoS One. 2010 Dec 31;5(12):e14478. doi: 10.1371/journal.pone.0014478 (PMC3013108; doi:10.1371/journal.pone.0014478)
Supplement: Table S2 — Adjusted and Unadjusted Regression Coefficients for Absolute Placenta Weight at Birth (Outcome), According to Life Stress and Emotional Symptoms During Pregnancy (Predictors) (N = 78017). Note. Life stress and emotional symptoms are continuous variables. CI = confidence interval. A Crude model provided in support of transparency. B Model adjusted for maternal age, infant sex, pre-pregnancy body mass index, parity, hypertension, gestational diabetes, and smoking. C To provide statistical values, which allow comparison of results between separate regression analyses, standardized regression coefficient estimates (beta) were calculated in addition to the unstandardized regression coefficient estimates (B). As the clustered variance estimation procedure does not provide betas, for illustrative purposes, betas were calculated with the robust variance estimation procedure. (0.03 MB DOC) [file pone.0014478.s002.doc]

**Supplemental Digital Content 2 (Table S2):
Adjusted and Unadjusted Regression Coefficients for Absolute Placenta Weight at Birth (Outcome), According to Life Stress and Emotional Symptoms During Pregnancy (Predictors) (*N* = 78017).**

|  | **Parameter estimates of the crude**^A^ **model** | | | **Parameter estimates of the adjusted**^B^  **model** | | |
| --- | --- | --- | --- | --- | --- | --- |
|  | ***B*** *[****95% CI for B****]* | ***Beta****^C^* | ***p*** | ***B*** *[****95% CI for B****]* | ***Beta****^C^* | ***p*** |
| **Placenta weight (g)** |  |  |  |  |  |  |
| Life stress | 2.75 [2.14, 3.36] | 0.037 | < 0.001 | 1.74 [1.13, 2.35] | 0.024 | < 0.001 |
| Emotional symptoms | –0.45 [–0.92, 0.01] | –0.008 | 0.054 | –0.28 [–0.73, 0.17] | –0.005 | 0.225 |
| *Crude Model: F(2,73174) = 43.12, P < 0.001, R^2^ = 0.001;*  *Adjusted Model: F(18, 73174) = 163.09, P < 0.001, R^2^ = 0.037* | | | | | | |
