# Supplementary material for: Maternal Psychosocial Stress during Pregnancy and Placenta Weight: Evidence from a National Cohort Study
Source: PLoS One. 2010 Dec 31;5(12):e14478. doi: 10.1371/journal.pone.0014478 (PMC3013108; doi:10.1371/journal.pone.0014478)
Supplement: Table S3 — Adjusted and Unadjusted Regression Coefficients for Placenta Weight at Birth, Corrected for Length of Gestation (Outcome)A, According to Life Stress and Emotional Symptoms During Pregnancy (Predictors), Stratified According to Socioeconomic Status (N = 78017). Note. Life stress and emotional symptoms are continuous variables. CI = confidence interval. SES = socioeconomic status. A As indicated by the gestational age- and sex-specific z-scores of placenta weight at birth. For convenience, the unstandardized regression coefficient estimates (B) (and their 95% CI) for z-standardized dependent variables are presented in [*10−3]. B Crude model provided in support of transparency. C Model adjusted for maternal age, infant sex, pre-pregnancy body mass index, parity, hypertension, gestational diabetes, and smoking. D To provide statistical values, which allow comparison of results between separate regression analyses, standardized regression coefficient estimates (beta) were calculated in addition to the unstandardized regression coefficient estimates (B). As the clustered variance estimation procedure does not provide betas, for illustrative purposes, betas were calculated with the robust variance estimation procedure. (0.04 MB DOC) [file pone.0014478.s003.doc]

**Supplemental Digital Content 3 (Table S3):
Adjusted and Unadjusted Regression Coefficients for Placenta Weight at Birth, Corrected for Length of Gestation (Outcome)^A^, According to Life Stress and Emotional Symptoms During Pregnancy (Predictors), Stratified According to Socioeconomic Status (*N* = 78017).**

|  | **Parameter estimates of the crude**^B^ **model** | | | **Parameter estimates of the adjusted**^C^  **model** | | |
| --- | --- | --- | --- | --- | --- | --- |
|  | ***B*** *[****95% CI for B****]* | ***Beta****^D^* | ***p*** | ***B*** *[****95% CI for B****]* | ***Beta****^D^* | ***p*** |
| **Placenta weight (ZS)** |  |  |  |  |  |  |
| *Life stress* | | | | | | |
| Low SES | 3.77 [-9.06, 16.60] | 0.008 | 0.565 | 2.33 [-10.29, 14.95] | 0.005 | 0.716 |
| Medium SES | 23.34 [16.01, 30.68] | 0.044 | < 0.001 | 15.19 [7.94, 22.45] | 0.029 | < 0.001 |
| High SES | 27.73 [21.43, 34.04] | 0.053 | < 0.001 | 17.34 [11.09, 23.60] | 0.032 | < 0.001 |
| *Emotional symptoms* | | | | | | |
| Low SES | -3.51 [-12.94, 5.91] | -0.011 | 0.465 | -0.66 [-9.99, 8.67] | -0.002 | 0.890 |
| Medium SES | -2.35 [-7.89, 3.19] | -0.006 | 0.406 | -0.62 [-6.09, 4.86] | -0.002 | 0.826 |
| High SES | 0.83 [-3.95, 5.60] | 0.002 | 0.735 | 0.96 [-3.77, 5.68] | 0.002 | 0.691 |
| *Low SES: Crude Model: F (2, 6203) = 0.29, P = 0.745, R^2^ < 0.001; adjusted model: F (18, 6203) = 13.90, P < 0.001, R^2^ = 0.040* | | | | | | |
| *Medium SES: Crude Model: F (2, 25846) = 23.12, P < 0.001, R^2^ = 0.002; adjusted model: F (18, 25846) = 61.73, P < 0.001, R^2^ = 0.041* | | | | | | |
| *High SES: Crude model: F (2, 36045) = 52.17, P < 0.001, R^2^ = 0.003; adjusted model: F (18, 36045) = 72.51, P < 0.001, R^2^ = 0.034* | | | | | | |
